# Antibiotic Resistance of *Helicobacter pylori* in Children with Gastritis and Peptic Ulcers in Mekong Delta, Vietnam

**DOI:** 10.3390/healthcare10061121

**Published:** 2022-06-17

**Authors:** Loan Thi Thuy Le, Tuan Anh Nguyen, Nghia An Nguyen, Yen Thi Hai Nguyen, Hai Thi Be Nguyen, Liem Thanh Nguyen, Mai Tuyet Vi, Thang Nguyen

**Affiliations:** 1 Department of Pediatrics, Can Tho University of Medicine and Pharmacy, Can Tho City 900000, Vietnam; 2 Department of Pediatrics, University of Medicine and Pharmacy at Ho Chi Minh City, Ho Chi Minh City 700000, Vietnam; nguyenanhtuan@ump.edu.vn (T.A.N.); nghianguyen@ump.edu.vn (N.A.N.); 3 Department of Microbiology, Can Tho University of Medicine and Pharmacy, Can Tho City 900000, Vietnam; nthyen@ctump.edu.vn (Y.T.H.N.); ntbhai@ctump.edu.vn (H.T.B.N.); 4 Faculty of Nursing and Medical Technology, Can Tho University of Medicine and Pharmacy, Can Tho City 900000, Vietnam; ntliem@ctump.edu.vn; 5 Department of Pharmacology and Clinical Pharmacy, Can Tho University of Medicine and Pharmacy, Can Tho City 900000, Vietnam; maivivi127@gmail.com

**Keywords:** children, antimicrobial resistance, eradication, *Helicobacter pylori*

## Abstract

Background: *Helicobacter pylori* (*H. pylori*) infection causes gastritis, duodenal and gastric ulcers, and gastric cancer. *H. pylori* eradication efficacy is low worldwide, and antibiotic resistance is the leading cause of therapy failure; therefore, this study was performed to determine the characteristics of antibiotic resistance of *H. pylori* in children with gastritis, duodenal and gastric ulcer. Methods: A cross-sectional study was conducted on 237 pediatric patients diagnosed with gastroduodenal inflammation and ulcer at two hospitals in Vietnam from March 2019 to April 2022. Pediatric patients with positive *H. pylori* tests continued to do E-tests to measure the minimum inhibitory concentration of the antibiotic so that we could prescribe effective antibiotics based on the sensitivity. Results: In 237 pediatric patients (51.1% males) with a median age of 10.3 years (range 5–16 years), endoscopic images showed that inflammatory lesions and peptic ulcers accounted for 69.2% and 30.8%, respectively. Resistance rates of *H. pylori* were 80.6% to clarithromycin (CLR), 71.7% to amoxicillin (AMX), 49.4% to metronidazole (MTZ), 45.1% to levofloxacin (LEV), and 11.4% to tetracycline (TET); dual resistance to AMX + CLR was 64.2%, AMX + LEV 35%, AMX + MTZ 33.3%, CLR + MTZ 32.5%, and TET + MTZ 7.2%. The frequency of clarithromycin resistance was significantly increased, particularly in pediatric patients who had received prior *H. pylori* treatment. The percentage of amoxicillin resistance increased with age; amoxicillin resistance of *H. pylori* was more prevalent among pediatric patients with peptic ulcers than those with gastroduodenal inflammation and higher in males than females. Conclusions: The proportions of resistance to CLR, AMX, MTZ, and LEV were extremely high, in contrast to TET, which was lower in pediatric patients. Our study suggests that the standard triple therapy with CLR should be limited as the empiric therapy for pediatric patients, and we should consider using eradication regimens with TET for children over 8 years of age if the medical facility is not qualified to perform antibiotic susceptibility tests of *H. pylori* in the Mekong Delta.

## 1. Introduction

*Helicobacter pylori (H. pylori)* is the leading cause of upper gastrointestinal diseases such as peptic ulcers, chronic gastritis, atrophic gastritis, and gastric cancer. About one-third of all children worldwide are infected with *H. pylori*, and its prevalence varies between regions of the world—low in developed countries and high in developing countries [1]. Triple therapy, including proton pump inhibitors (PPIs), amoxicillin, and clarithromycin or metronidazole, has low eradication efficacy [2]. Domestic and abroad studies indicate that antibiotic resistance to *H. pylori* has risen, which is the main reason why the effectiveness of the standard regimen has gradually decreased below 80%.

The antibiotics used to treat *H. pylori* eradication in children are limited. For an extended period, the standard triple therapy (PPI–amoxicillin–clarithromycin or metronidazole) has been the first-line therapy recommended by international guidelines to eradicate *H. pylori* infection. Tetracycline and levofloxacin are only allowed for eradicating *H. pylori* infection in adults. However, among pediatric patients, tetracycline is only indicated for children >8 years of age, and levofloxacin is only allowed in adolescents because of some adverse effects on the development of teeth and cartilage. Therefore, treating *H. pylori* is becoming a big challenge for pediatric clinicians. Based on the recent recommendation of ESPGHAN/NASPGHAN in 2017, initial eradication treatment should be based on the antibiotic sensitivity of *H. pylori*. The eradication rate should be ≥90% to prevent *H. pylori* from developing secondary resistant strains and the spread of antibiotic-resistant strains in the community, reduce the costs and risks of rescue therapy, and, ultimately, prevent and decrease the incidence of gastric cancer [2,3]. Nevertheless, taking the *H. pylori* test faces obstacles because this test is only completed in central laboratories. The expensive test charge is also a problem. The evidence of antibiotic resistance in each geographic area plays an essential role in choosing empiric regimens. Therefore, we conduct this study to evaluate the susceptibility of *H. pylori* to some current antibiotics in order to have the necessary basis for selecting appropriate regimens.

## 2. Materials and Methods

### 2.1. Setting and Study Design

The sample size was estimated by using the above formula:

$$n=\frac{Z^{2}{}_{1-\alpha /2}\;x\;P\left(1-P\right)}{d^{2}}$$

According to the recommendations of ESPGHAN/NASPGHAN and the Ministry of Health of Vietnam, clarithromycin (CLR), amoxicillin (AMX), metronidazole (MTZ), levofloxacin (LEV), and tetracycline (TET) are allowed for use in eradication treatment in children. However, the spread of AMX- and/or CLR-resistant *H. pylori* is one of the significant causes of treatment failure in children. Therefore, we calculated the sample size based on the estimated rate of AMX- and CLR-resistant *H. pylori* from a prior study by Quek C. (2016), conducted on pediatric patients with gastritis and peptic ulcers (12.5% and 84.6%, respectively) from Ho Chi Minh city [4]. There was an assumed margin of error of 5% and a confidence level of 95%, resulting in 169 patients with AMX-resistant *H. pylori* and 201 patients with CLR-resistant *H. pylori.* To minimize the error, the actual sample size was 237 samples.

A cross-sectional study was carried out in Can Tho Children’s Hospital and Can Tho University of Medicine and Pharmacy in Vietnam from March 2019 to April 2022. The sample size included 237 pediatric patients aged 5 to 15 years who presented with gastrointestinal symptoms, had indications for esophagogastroduodenoscopy (EGD), and had a positive *H. pylori* culture test. The exclusion criteria included pediatric patients who had a history of gastric bypass surgery, had gastrointestinal bleeding, used antibiotics or bismuth within 4 weeks before the endoscopy, used PPIs within 2 weeks before the endoscopy, or had a history of allergy to one of the drugs in the study therapy.

EGD was performed after the patient was completely anesthetized by well-trained endoscopists at the Endoscopy Center of Can Tho University of Medicine and Pharmacy and the Gastrointestinal Endoscopy unit of the Gastroenterology Department of Can Tho Children’s Hospital. During upper gastrointestinal endoscopy, we collected 4 gastric mucosa biopsies. One biopsied piece at the gastric antrum and one at the gastric body were taken initially for *H. pylori* culture; these biopsies were placed in a transportation medium and immediately transferred to the Department of Microbiology, Can Tho University of Medicine and Pharmacy, for culturing. One biopsied piece at the gastric antrum and one at the gastric body were for the urease test (NK Pylori test, Nam Khoa Biotek Co., Ltd., Ho Chi Minh City, Vietnam).

We selected 5 types of antibiotics: clarithromycin (CLR), amoxicillin (AMX), metronidazole (MTZ), levofloxacin (LEV), and tetracycline (TET). Because these antibiotics were allowed for use in eradication treatments in children, according to the recommendations of ESPGHAN/NASPGHAN and the Ministry of Health in Vietnam, this study also evaluated dual resistance with AMX–CLR, AMX–MTZ, AMX–LEV, CLR–MTZ, and TET–MTZ to help clinicians see the risk of failure in eradicating *H. pylori* when choosing the corresponding empiric regimen.

### 2.2. Helicobacter pylori Culture and Antimicrobial Susceptibility Testing

Biopsies taken at gastric antrum and body by gastroduodenoscopy were used for the process of *H. pylori* culture. Biopsy fragments were added to 500 µL of transportation medium (20% glycerol, 0.9% NaCl in Milli-Q water). Then, the biopsy fragments were ground in a culture medium (100 µL of Brain Heart Infusion (BHI) solution supplemented with 10% fetal bovine serum (FBS)). The following step was to culture the fragments on an agar plate supplemented with 10% lysed sheep blood (Nam Khoa Biotek Co., Ltd.), 1% isoVitale, a skin antibiotic mixture, and amphotericin B. The agar plates were incubated at 37 °C in a specific microaerobic atmosphere (mixture of O_2_:CO_2_:N_2_ gas at the ratio of 5:10:85, respectively) for 4–5 days. A single colony in a culture medium for 4–5 days was determined based on colony morphology and the features of *H. pylori,* including Gram-negative S-shaped bacterium and being urease-positive, oxidase-positive, and catalase-positive.

Minimal inhibitory concentrations (MICs) of 5 different antibiotics, namely, amoxicillin (AMX), clarithromycin (CLR), levofloxacin (LEV), tetracycline (TET), and metronidazole (MTZ), were determined by E-test (BioMerieux, Marcy-l’Étoile, France). According to the 2019 standards of the European Committee on Antimicrobial Susceptibility (EUCAST) to evaluate susceptibility, the resistance cutoff values were 0.125 µg/mL for amoxicillin, 0.5 µg/mL for clarithromycin, 1 µg/mL for levofloxacin and tetracycline, and 8 µg/mL for metronidazole [5].

### 2.3. Statistical Analysis

Data were analyzed using the Statistical Package for Social Sciences (SPSS) version 20.0. Descriptive statistical analysis was used to describe the characteristics of the pediatric patients, such as gender, age, residency, gastric disease, and susceptibility to 5 antibiotics of the strains isolated from the clinical samples. A chi-square test was used to correlate the difference between proportions. Fisher’s exact test was used when more than 20% of the expected counts were less than 5. A *p*-value less than 0.05 was accepted as statistically significant.

## 3. Results

### 3.1. Patient Characteristics

Regarding the 237 children recruited for this study, 48.9% were males and 52.1% were females. The mean age of the pediatric patients was 10.03 ± 2.53 years, ranging from 5 to 16; their ages followed a normal distribution. The vast majority of patients came from Can Tho city (63.3%), which was significantly higher than patients coming from the provinces located along the Mekong River (Vinh Long, Hau Giang, Soc Trang, Dong Thap, Tien Giang, Ben Tre, An Giang, etc.) (36.7%). Almost one-third of the pediatric patients were diagnosed with peptic ulcer (30.8%), and 69.2% were diagnosed with nodular gastritis/duodenitis; 77.2% of strains were isolated from pediatric patients without previous therapy (Table 1).

### 3.2. Antibiotic Resistance

**Figure 1:**
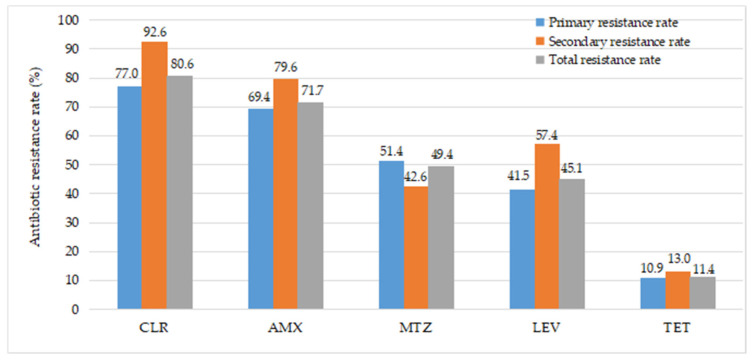
The frequency of *H. pylori* antibiotic resistance in Vietnamese pediatric patients. CLR, clarithromycin; AMX, amoxicillin; MTZ, metronidazole; LEV, levofloxacin; TET, tetracycline.

The percentage of average resistance to clarithromycin, amoxicillin, metronidazole, levofloxacin, and tetracycline were 80.6% (191/237), 71.7% (170/237), 49.4% (117/237), 45.1% (107/237), and 11.4% (27/237), respectively. The primary resistance prevalence was 77.0% (141/183) for CLR, 69.4% (127/183) for AMX, 41.5% (76/183) for LEV, 51.4% (94/183) for MTZ, and 10.9% (20/183) for TET. Of the 54 strains isolated from patients prior H. pylori treatment, the secondary resistance prevalence was 92.6% (50/54) for CLR, 79.6% (43/54) for AMX, 57.4% (31/54) for LEV, 42.6% (23/54) for MTZ, and 13.0% (7/54) for TET (Figure 1).

Among 237 *H. pylori* strains from pediatric patients, no strains were sensitive to all five antibiotics; 10.1% (24/237) had mono resistance, 32.9% (78/237) had dual resistance, 46.8% (111/237) had triple resistance, 8.9% (21/237) had quadruple resistance, and 1.3% (3/237) had resistance to all five antibiotics. The remaining 135 strains were resistant to three or more antibiotics; thus, multidrug resistance accounted for 57.0% (Table 2).

**Figure 2:**
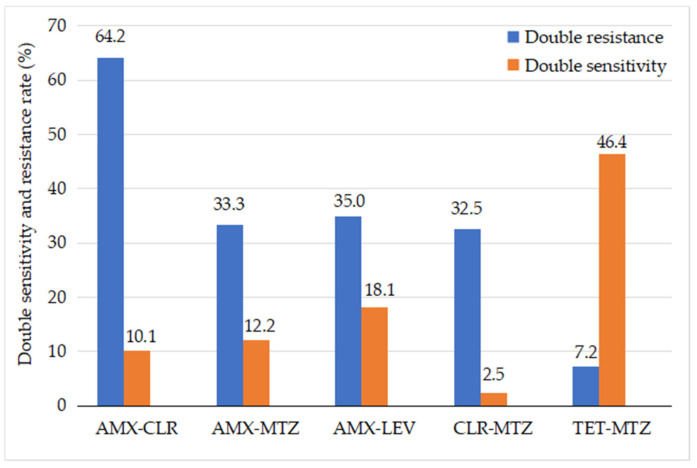
The frequency of double susceptibility and resistance to antibiotics of *H. pylori* in pediatric patients. CLR, clarithromycin; AMX, amoxicillin; MTZ, metronidazole; LEV, levofloxacin; TET, tetracycline.

Analysis of antibiotic sensitivity in *H. pylori* bacteria to select an effective eradication therapy found that double resistance to AMX + CLR was 64.2% (148/237), and susceptibility to both was 10.1% (24/237); double resistance to AMX + MTZ was 33.3% (79/237), and susceptibility to both was 12.2% (29/237); double resistance to AMX + LEV was 35% (83/237), and susceptibility to both was 18.1% (43/237); double resistance to CLR + MTZ was 32.5% (77/237), and susceptibility to both was 2.5% (6/237); double resistance to TET + MTZ was 7.2% (17/237), and susceptibility to both was 46.4% (110/237) (Figure 2).

### 3.3. Factors Associated with Antibiotic Resistance

The frequency of CLR resistance was significantly increased in the group of children previously treated with *H. pylori*. In particular, the rate of CLR resistance in the group of children who had received prior *H. pylori* eradication treatment was 92.6%. In comparison, the resistance rate in the group of non-treatment children was 77.0% (*p* = 0.01). The percentage of AMX resistance increased with age (88.1%/11–16 years old group compared to 55.5%/5–10 years old group (*p* = 0.00)), and the resistance rate was higher in males than females (79.3% compared to 64.6%, *p* = 0.01). The AMX resistance of *H. pylori* was more prevalent among pediatric patients with peptic ulcers than those with gastroduodenal inflammation. There was no difference in the frequency of *H. pylori* resistance to MTZ and LEV by sex, age group, history of previous *H. pylori* treatment, and lesions on gastroduodenal endoscopy (Table 3).

## 4. Discussion

Several factors affected the effectiveness of eradication treatment, but antibiotic resistance of *H. pylori* was the main cause of treatment failure. In the past 20 years, the rate of resistance of *H. pylori* to some prevalent antibiotics used in eradication therapy has tended to increase. One of the main contributors to this trend is the consequence of the prescription of antibiotics for other infectious diseases and the widespread consumption of antibiotics among the population in Vietnam [5]. In this study, the overall resistance rates to AMX, CLR, MTX, LEV, and TET were 71.7%, 80.6%, 49.4%, 45.1%, and 11.4%, respectively. The proportion of CLR resistance of *H. pylori* in this study (80.6%) was higher than in other worldwide studies, such as Ogata S.K.’s study on Brazil (19.5%), Krzyzek P.’s study on Poland (54.5%), Silva G.M.’s study on Portugal (23.3%), and Li J.’s study on China (55.2%) [6,7,8,9]. Comparing previous studies on Vietnam, the rate of resistance to CLR has been increasing, for instance, 50.9% in 2006 [10], 84.6% in 2016 [4], 92.1% in 2021 [11], and 81% in 2022 (in this study). Generally, the percentage of pediatric patients infected with CLR-resistant strains of *H. pylori* in different continents is higher than 20% [6,7,8,9,10,11,12,13,14,15,16]. However, in recent decades, the affordable charge for antibiotics and improperly controlled antibiotic use in Vietnam is why CLR has been widely used, especially in children with respiratory infections. Hence, *H. pylori* bacteria are less sensitive to CLR. Unfortunately, many previous studies have found that the susceptibility of *H. pylori* to CLR in vitro is closely related to its ability to eradicate this bacterium clinically. In a meta-analysis of 10,178 patients, Fischbach L.A. showed that when pediatric patients were treated with a standard regimen (PPI, AMX, and CLR), resistance to CLR was significantly reduced to 66.2% (95% CI 58.2–74.2), and the treatment effectiveness and prediction of successful eradication of *H. pylori* with this regimen ranged from 0–50% [17]. Tancovic J. found similar eradication efficiency, which decreased from 79% in patients infected with CLR-sensitive *H. pylori* chains to 12% in the CLR-resistant group [18]. Therefore, prescribing CLR in *H. pylori* eradication regimens for pediatric patients should be recommended when there is evidence of *H. pylori* susceptibility to CLR.

The AMX resistance of *H. pylori* is one of the most significant concerns for clinicians as most eradication regimens include AMX. The resistance rate to AMX detected in this study (73%) was higher than in previous studies [6,7,8,9,10,11,13,14,15,16]. Although studies might differ in geographical factors, sample size, duration of the study, and antimicrobial testing methods, the prevalence of *H. pylori* resistance to AMX is increasing. This is a significant threat that affects the success of eradication regimens with AMX. The high prevalence of AMX resistance in our study could be explained by the combination of amoxicillin with clavulanate potassium in the empiric therapy commonly prescribed for many infectious diseases. In addition, Vietnam is a country where AMX can be purchased without a prescription, resulting in unsupervised antibiotic use in the treatment of infectious conditions in children.

Several reports have recorded that the percentage of *H. pylori* MTZ resistance ranges from 3.3% to 86%; the statistics were mainly gathered from Asian countries, excluding Japan [6,7,8,9,10,11,12,13,14,15,16]. Meanwhile, the effect of MTZ resistance on the efficacy of eradication treatments was lower than that of CLR and LEV resistance. The MTZ resistance of *H. pylori* was poorly associated with eradication efficacy. According to the WHO, pediatric patients infected with *H. pylori* chains that are resistant to CLR were 7 times more likely to have treatment failure than those susceptible to CLR (OR = 7; 95% CI, 5.23–9.28; *p* < 0.001); this was similar for LEV (8.2 times); dual resistance to CLR and MTZ was 9.4 times and only 2.5 times for mono resistance to MTZ (95% Cl, 1.82–3.48; *p* = 0.004) [19,20]. The frequency of MTZ resistance in the population was approximately 30%, which would reduce the overall efficacy of eradication by 17–18% [21]. With these data, clinicians might consider prescribing MTZ in some cases of MTZ resistance of *H. pylori* in vitro. This plays an essential role in pediatric clinical practice because of the age limits for antibiotic use. Furthermore, high-dose MTZ (1.5–2 g per day in adults), prolonged treatment, or combinations with PPIs or bismuth had the capacity to resolve the MTZ resistance of *H. pylori* [19,21,22]. However, the use of high-dose MTZ might cause poor gastrointestinal tolerance and other undesirable effects. A helpful way to reduce these adverse effects for pediatric patients is to divide the drug into 3–4 doses a day, take it after meals, and avoid drinking alcohol and beer during treatment.

In addition, our study recorded a high rate of *H. pylori* resistance to LEV in pediatric patients (41%) because quinolones are licensed for use in pediatric diarrhea in Vietnam and children have inherited LEV-resistant strains of *H. pylori* from their parents. There have been few studies on the susceptibility of *H. pylori* to LEV in children. The overview by Chen P.Y., performed on 4574 adult patients from 322 studies, showed that the eradication rate in LEV-sensitive *H. pylori* strains reached 81.1%, while it was only 36.3% in resistant strains. These studies also discouraged using 3-drug regimens with LEV if the percentage of *H. pylori* resistance was over 5–10% [23]. Megraud F.’s study noticed that fluoroquinolones should only be prescribed to patients who had never been exposed to this antibiotic in *H. pylori* eradication [24]. However, ESPGHAN/NAPGHAN suggests only prescribing LEV for eradication in adolescents [2]. Furthermore, the figure for overall *H. pylori* resistance to TET was relatively low (11.4%), similar to global reports [6,7,8,9,12,13,14,15,16]. However, TET is only used in pediatric patients over 8 years old due to the side effects on teeth and bone.

Finally, in terms of the treatment of infectious diseases, successful treatment largely depends on the types of prescribed antibiotics to which the bacteria are susceptible. Treatment of *H. pylori* is similar to tuberculosis treatment, another difficult-to-treat infectious disease that requires several antibiotics simultaneously. Moreover, evaluating the antibiotic sensitivity of *H. pylori* bacteria supports the selection of an effective treatment regimen. The study found that the treatment failure of the combination therapy of AMO and CLR was highest (64.2%), followed by AMX and LEV (35%), AMX and MTZ (33.3%), and CLR and MTZ (32.5%); the lowest rate was for the TET and MTZ combination therapy (7.2%). According to the 2017 Maastricht V consensus, in regions with a high prevalence of *H. pylori* dual resistance to CLR and MTZ, the standard 3-drug regimen (PPI–AMX–CLR, PPI–AMX–MTZ) cannot be used as a first-line regimen; 4-drug therapy with bismuth is a better option [25]. This is a challenge for clinicians in treating *H. pylori* in children because their patients must take medicine four times a day (before and after breakfast, before and after lunch, before and after dinner, and at night before bed). Hence, doctors should educate children and their family members to strengthen treatment adherence and to avoid poor adherence or quitting treatment, which causes treatment failure. Moreover, the use of TET antibiotics for pediatric patients is limited by age, in general, and the eradication of *H. pylori* in children ≤8 years of age may encounter many obstacles. According to the recommendations of ESPGHAN/NASPGHAN (2017) and JSPGHAN (2020), eradication therapy should be based on the susceptibility of *H. pylori* to antibiotics, possibly determined by an antibiogram test (E-test or dilution method on agar) or molecular biology tests (real-time PCR or fluorescence in situ hybridization) [2,26].

The weakness of our study is that due to cost issues, we have not been able to perform genetic sequencing to determine the number of *H.* *pylori* strains that infected our patients. Therefore, in a few cases of infection with multiple strains of *H.* *pylori*, antibiotic sensitivity results may not have been accurate for those patients.

## 5. Conclusions

The study found that antibiotic resistance rates of *H. pylori* to CLR, AMX, MTZ, and LEV are extremely high, whereas TET resistance is low in pediatric patients. We suggest that the standard triple therapy with CLR should be restricted as empiric therapy for pediatric patients based on antimicrobial susceptibility profiles. Due to the limited choice of antibiotics for *H. pylori* treatment in patients younger than 8 years old, doctors should only indicate *H. pylori* eradication when necessary. Bismuth quadruple therapy is suitable as the first-line regimen for patients older than 8 years if they do not have access to antimicrobial susceptibility tests for *H. pylori* in the Mekong Delta.

## Figures and Tables

**Table 1:** Patient characteristics.

Demographic Characteristics	Frequency (*n* = 237)	Percentage (%)
Gender		
Male	116	48.9
Female	121	51.1
Age		
Mean age	10.03 ± 2.53	
5–8 years	60	25.3
9–12 years	122	51.5
13–16 years	55	23.2
Geographic area		
Can Tho city	150	63.3
Nearby regions	87	36.7
Endoscopy findings		
Nodular Gastritis/duodenitis	168	69.2
Gastric ulcer	5	2.1
Duodenal ulcer	68	28.7
History eradication		
Without previous therapy	183	77.2
Prior to *H. pylori* treatment	54	22.8

**Table 2:** The proportion of multidrug resistance to antibiotic agents in pediatric patients.

Antibiotic Resistance	Frequency (*n* = 237)	Percentage (%)
Mono resistance	24	10.1
Dual resistance	78	32.9
Triple resistance	111	46.8
Quadruple resistance	21	8.9
All resistance	3	1.3
Multidrug resistance	135	57.0

**Table 3:** Factors associated with antimicrobial resistance to CLR, AMX, MTZ, and LEV.

Factors	Overall	CLR-R	*p*	AMX-R	*p*	MTZ-R	*p*	LEV-R	*p*
		*n* (%)		*n* (%)		*n* (%)		*n* (%)	
Age									
5–10 years	119	92 (77.3%)	0.20	66 (55.5%)	0.00	58 (48.9%)	0.85	56 (47.1%)	0.55
11–16 years	118	99 (83.9%)		104 (88.1%)		59 (50.0%)		51 (43.2%)	
Gender									
Male	116	94 (81.0%)	0.87	92 (79.3%)	0.01	57 (49.1%)	0.95	49 (42.2%)	0.38
Female	121	97(80.2%)		78 (64.5%)		60 (49.6%)		58 (47.9%)	
Prior treatment									
No	183	141 (77.0%)	0.01	127 (69.4%)	0.14	94 (51.4%)	0.26	76 (41.5%)	0.39
Yes	54	50 (92.6%)		43 (79.6%)		23 (42.6)		31 (57.4%)	
EGD Findings									
Nodular Gastritis	164	127 (77.4%)	0.66	107 (65.2%)	0.00	76 (46.3%)	0.58	80 (48.8%)	0.79
Peptic ulcer	73	64 (87.7%)		63 (86.3%)		31 (42.5%)		37 (50.7%)	

CLR-R, clarithromycin-resistant; AMX-R, amoxicillin-resistant; MTZ-R, metronidazole-resistant; LEV-R, levofloxacin-resistant; TET-R, tetracycline-resistant.

## Data Availability

Not applicable.
